# A Phase I clinical trial of EUS-guided intratumoral injection of the oncolytic virus, HF10 for unresectable locally advanced pancreatic cancer

**DOI:** 10.1186/s12885-018-4453-z

**Published:** 2018-05-25

**Authors:** Yoshiki Hirooka, Hideki Kasuya, Takuya Ishikawa, Hiroki Kawashima, Eizaburo Ohno, Itzel B. Villalobos, Yoshinori Naoe, Toru Ichinose, Nobuto Koyama, Maki Tanaka, Yasuhiro Kodera, Hidemi Goto

**Affiliations:** 1 Department of Endoscopy, Nagoya University Hospital, 65 Tsuruma-cho, Showa-ku, Nagoya, 466-8550 Japan; 2 Cancer Immune Therapy Research Center, Nagoya University Graduate School of Medicine, Nagoya, Japan; 3 Department of Gastroenterology, Nagoya University Graduate School of Medicine, Nagoya, Japan; 4 Takara Bio Inc., Otsu, Japan; 5 Department of Surgery II, Nagoya University Graduate School of Medicine, Nagoya, Japan

**Keywords:** Pancreatic cancer, Oncolytic virus, HF10, EUS-guidance

## Abstract

**Background:**

Prognosis of pancreatic cancer is poor with a 5-year survival rate of only 7%. Although several new chemotherapy treatments have shown promising results, all patients will eventually progress, and we need to develop newer chemotherapy treatments to improve response rates and overall survival (OS). HF10 is a spontaneously mutated oncolytic virus derived from a herpes simplex virus-1, and it has potential to show strong antitumor effect against malignancies without damaging normal tissue. We aimed to evaluate the safety and anti-tumor effectiveness in phase I dose-escalation trial of direct injection of HF10 into unresectable locally advanced pancreatic cancer under endoscopic ultrasound (EUS)-guidance in combination with erlotinib and gemcitabine administration. The mid-term results have been previously reported and here we report the final results of our study.

**Methods:**

This was a single arm, open-label Phase I trial. HF10 was injected once every 2 weeks and continued up to four times in total unless dose-limiting toxicity (DLT) appears. A total of nine subjects in three Cohorts with dose-escalation were planned to be enrolled in this trial. The primary endpoint was the safety assessment and the secondary endpoint was the efficacy assessment.

**Results:**

Twelve patients enrolled in this clinical trial, and ten subjects received this therapy. Five patients showed Grade III myelosuppression and two patients developed serious adverse events (AEs) (perforation of duodenum, hepatic dysfunction). However, all of these events were judged as AEs unrelated to HF10. Tumor responses were three partial responses (PR), four stable diseases (SD), and two progressive diseases (PD) out of nine subjects who completed the treatment. Target lesion responses were three PRs and six SDs. The median progression free survival (PFS) was 6.3 months, whereas the median OS was 15.5 months. Two subjects from Cohort 1 and 2 showed downstaging and finally achieved surgical complete response (CR).

**Conclusions:**

HF10 direct injection under EUS-guidance in combination with erlotinib and gemcitabine was a safe treatment for locally advanced pancreatic cancer. Combination therapy of HF10 and chemotherapy should be explored further in large prospective studies. Trial registration: This study was prospectively registered in UMIN-CTR (UMIN000010150) on March 4th, 2013.

**Electronic supplementary material:**

The online version of this article (10.1186/s12885-018-4453-z) contains supplementary material, which is available to authorized users.

## Background

The number of death due to pancreatic cancer has been increasing and now it is the fourth leading cause of cancer mortality in the United States with a 5 year survival rate of 7% [1], which was similar in Japanese population [2]. Surgery offers the only chance for cure, but most of the patients present with advanced stage and only 15–20% of those are candidates for curative resection [3, 4]. Chemotherapy may play a more important role in the treatment of advanced or inoperable pancreatic cancer. Although the appearance of several new chemotherapy treatments has shown improving survival, all patients will eventually progress and die of the disease. Therefore, we need to develop novel anti-cancer treatments to achieve further improvement of prognosis.

Because of their distinctive characteristics in replication and antitumor immune responses, Oncolytic viruses (OVs) are considered to be a new option in cancer therapy. Most of the OVs developed in the past have been generated to increase the tumor selectivity and efficacy. However, in contrast to those artificially modified OV mutants, HF10 is a spontaneously mutated OV derived from a herpes simplex virus-1 (HSV-1). Genetically, HF10 naturally lacks the expression of *UL43*, *UL49.5*, *UL55*, *UL56*, and latency-associated transcripts, and overexpresses *UL53* and *UL54* [5]. Although the effect of these genetic changes are still under investigation, based on the previous studies, the characteristics of HF10 can be summarized into following five points: 1. high tumor selectivity, 2. high viral replication, 3. initiation of a cytopathic effect, 4. intermediation of potent bystander effect, and 5. strong antitumor effect against various malignancies [5]. In addition, it has been reported that lack of UL 56 expression may reduce the neuroinvasiveness [6]. Following these results, successful clinical trials with promising results have been reported in different cancer types including recurrent metastatic breast cancer [7, 8], recurrent head and neck squamous cell carcinoma (HNSCC) [9], unresectable pancreatic cancer [10], refractory and superficial cancers [11], and melanoma [12].

Up to now, OVs have not shown serious toxicities or any therapeutic resistance, contrary to chemotherapeutic drugs that may cause severe dose limiting toxicities (DLT). As OVs and chemotherapeutic drugs have different mechanisms of action each other, combination therapy is expected to increase the antitumor effect with limited side effects. Although the data on HF10 in preclinical and clinical trials suggest that therapeutic applications can be developed with a high safety margin, ideal combination therapies with either chemotherapy or immunotherapeutic agents still need more investigation [5]. In this study, we conducted the phase I dose-escalation trial of HF10 direct injection into unresectable locally advanced pancreatic cancer under EUS-guidance in combination with erlotinib and gemcitabine administration. We assessed the safety and antitumor effectiveness of a novel triple combination therapy. The mid-term results have been previously reported [13] and here we report the final results of our study.

## Methods

### Study design

This was a single arm, open-label Phase I trial. This study was registered in UMIN-CTR (UMIN000010150) and was approved by the Ethical Committee in our institute. Written informed consents to participate were obtained from all the patients in this study.

The following inclusion criteria were used for the selection of the patients: 1) Patients diagnosed with pancreatic cancer histopathologically through EUS-guided fine needle aspiration (EUS-FNA) and considered as locally advanced unresectable without distant metastasis (including non-regional lymph node metastasis) after discussion with surgical department based on NCCN Clinical Practice Guidelines in Oncology [14] (Additional file 1: Table S1); 2) Accessible for injection of HF10 under EUS-guidance; 3) At least one measurable lesion according to Response Evaluation Criteria in Solid Tumors (RECIST) criteria; 4) Eastern Cooperative Oncology Group (ECOG) performance status (PS) of 0–2; 5) Estimated life expectancy of more than 3 months; 6) Older than 20 years and younger than 80 years of age; 7) adequate bone marrow function (white blood cell count≧4000/mm^3^, neutrophil count≧2000/mm^3^, platelet count≧100,000/mm^3^); 8) Adequate renal function (creatinine clearance (Cockroft-Gault Equation)≧60 ml/min); 9) Adequate liver function (serum total bilirubin≦2 times the upper limits of normal (ULN), transaminases≦1.5 times ULN); 10) Patients who provided written informed consent; 11) Positive HSV-1 antibody.

The exclusion criteria were as follows: Bleeding diathesis; Ascites, pleural effusion, cardiac effusion to be treated; Active infection; Duplicated active cancers (synchronous duplicated cancer or metachronous cancer with less than 5 years of disease free period); Increased intracranial pressure to be treated due to brain metastases; pregnant or lactating women; Allergic to live vaccine; Use of anti HSV drugs; Implementation of immunotherapy for cancer; Positive HBs antigen, HCV antibody or HIV antibody; Adrenal insufficiency, hemodialysis, unilateral kidney.

### Treatment

**Fig. 1:**
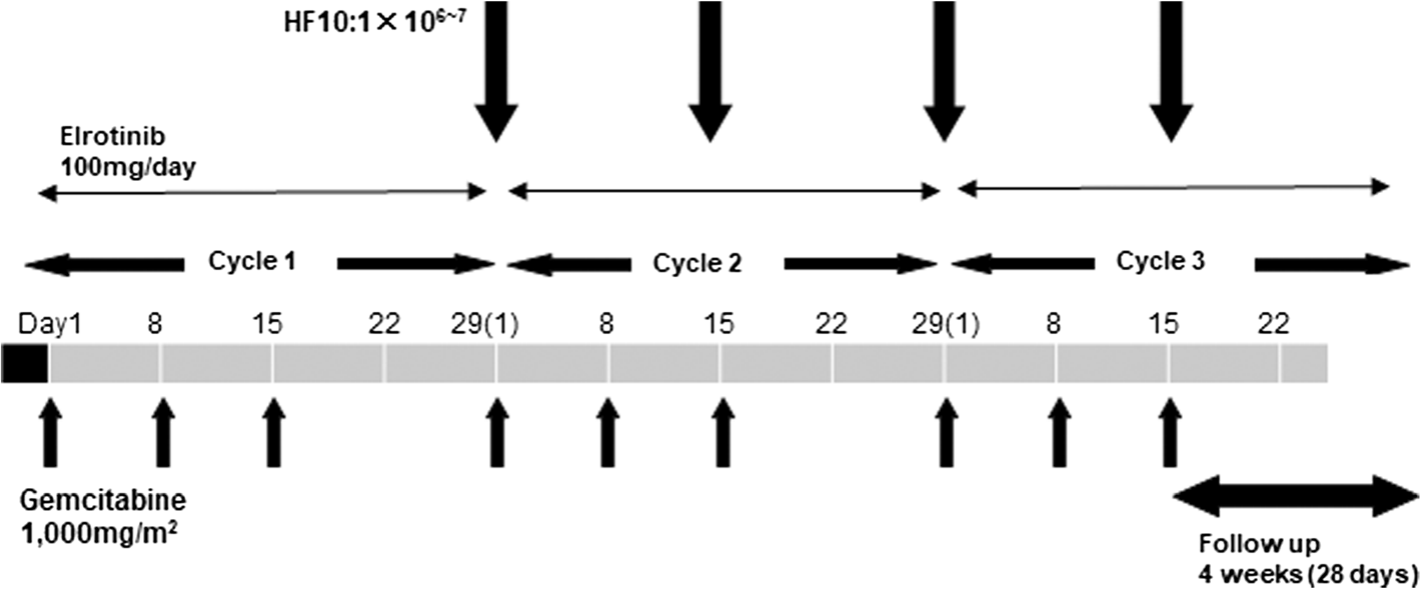
Schedule of the treatment

Following one cycle of erlotinib and gemcitabine therapy (gemcitabine 1000 mg/m2 weekly for 3 weeks, followed by a 1-week rest; erlotinib 100 mg orally, once daily), those judged tolerable for next cycle were final candidates. HF 10 injection was started at day 1 of cycle 2. The number of HF10 injection under EUS-guidance was to be four times in total (once every 2 weeks) unless DLT appears. DLT was defined as non-hematological toxicity higher than grade III according to the Common Terminology Criteria for Adverse Events, version 4.0 (CTCAE v4.0), febrile neutropenia or thrombocytopenia requiring transfusion. Three Cohorts, a total of nine subjects were planned to be enrolled in this trial [Cohort 1 (1 × 10^6^ pfu/day × 4 times): three subjects, Cohort 2 (3 × 10^6^ pfu/day x 4times): three subjects, Cohort 3 (1 × 10^7^ pfu/day x 4times): three subjects] (Fig. 1). If there was no DLT in the first three cases of each Cohort, the trial was proceeded with the next Cohort until the maximum tolerated dose (MTD) was determined. If one out of three cases showed DLT, three additional cases were registered for the same Cohort. If there was no DLT in the additional three cases, the trial was proceeded with the next Cohort. If one of them showed DLT, the dose was not increased considering it exceeded the MTD. If two out of three cases showed DLT, no more cases were added.

Injection of HF10 was performed under EUS-guidance to deliver the virus into the tumor.

### Assessments

The primary endpoint was the safety assessment (frequency and degree of toxicity). The adverse events (AEs) were graded according to the Common Terminology Criteria for Adverse Events, version 3.0 (CTCAE v3.0). A Safety Evaluation Committee was periodically held during the study and the correlation between HF 10 injection and AEs was discussed. The secondary endpoint was the efficacy assessment (complete response (CR), partial response (PR), stable disease (SD), and progressive disease (PD)), which was done by a computed tomography (CT) scan, which was performed at least once every 4 weeks according to the RECIST criteria. A CR was defined as the disappearance of all known disease determined by two observations not less than 3 weeks apart. A CR achieved after surgical approach was defined as surgical CR. A PR was defined as at least a 30% decrease in measurable disease by two observations not less than 3 weeks apart and no evidence of any new lesions or progression of any existing lesions. An inability to demonstrate a 30% decrease in tumor size or a 20% increase in the size of one or more lesions, as well as no new lesions for more than 6 weeks, was defined as SD. A 20% increase in the size of one or more measurable lesions or the appearance of any new lesions was defined as PD. The progression free survival (PFS) was measured from the date of enrollment until the date of PD. The overall survival (OS) was calculated from the date of enrollment until the date of death.

## Results

### Safety assessment

From June 2013 to May 2015, 12 patients were enrolled in the study. Two cases were excluded prior to the HF10 injection due to interstitial pneumonia and lymph node metastases after one cycle of erlotinib and gemcitabine therapy. Ten subjects including one dropout subject received this therapy (Table 1). Five of ten subjects showed myelosuppression (Grade III) caused by chemotherapy. Two of ten subjects developed serious AEs. One case developed perforative peritonitis following duodenal stenosis. Another case developed Grade IV hepatic dysfunction 1 week after the third injection of HF10, and the treatment was discontinued at this point (Table 2). All of these events were judged as AEs unrelated to HF10. There was no complication related to EUS or EUS-guided injection of HF10.

**Table 1:** Patients profiles

Patient no.		Age	Contents (p.f.u) X Time	Injection site	Staging^a^ (radiological)	Staging^a^ (postoperative)
Cohort 1	HF-1-02	60s	1X10^6^X4	Pancreas head	III (T4N0M0)	NA
	HF-1-04	60s	1X10^6^X4	Pancreas head (uncinate process)	III (T4N0M0)	NA
	HF-1-05	60s	1X10^6^X4	Pancreas body	III (T4N0M0)	IIA (T3N0M0)
Cohort 2	HF-2-01	60s	3X10^6^X4	Pancreas body	III (T4N0M0)	NA
	HF-2-02	60s	3X10^6^X4	Pancreas head (uncinate process)	III (T4N0M0)	IIA (T3N0M0)
	HF-2-03	60s	3X10^6^X4	Pancreas body	III (T4N0M0)	NA
Cohort 3	HF-3-01	60s	1X10^7^X3	Pancreas body	III (T4N0M0)	NA
	HF-3-02	50s	1X10^7^X4	Pancreas head (uncinate process)	III (T4N0M0)	NA
	HF-3-03	60s	1X10^7^X4	Pancreas body	III (T4N0M0)	NA
	HF-3-04	60s	1X10^7^X4	Pancreas head	III (T4N0M0)	NA

^a^Based on NCCN Clinical Practice Guidelines in Oncology [13]

**Table 2:** Safety evaluation

Patient no.		Toxicity of HF10	DLT	Evaluation (CTCAE ver 4.0)
Cohort 1	HF-1-02	(−)	(−)	Grade III Neutrophil and Platlet count decrease, Duodenal stenosis, Perforative peritonitis
	HF-1-04	(−)	(−)	Grade II fever, Blood bilirubin increase (stent failure), Interstitial pneumonia (After treatment)
	HF-1-05	(−)	(−)	NA
Cohort 2	HF-2-01	(−)	(−)	Grade III Neutrophil decrease
	HF-2-02	(−)	(−)	Grade III Neutrophil decrease
	HF-2-03	(−)	(−)	Grade III Neutrophil decrease and Grade II ALT increase
Cohort 3	HF-3-01	(−)	(−)	Grade IV Hepatobiliary disorder
	HF-3-02	(−)	(−)	NA
	HF-3-03	(−)	(−)	NA
	HF-3-04	(−)	(−)	Grade III Neutrophil decrease

*DLT* dose-limiting toxicity

### Efficacy assessment

Nine subjects who completed four injections of HF10 were included for the efficacy assessment.

Overall responses were three PRs, four SDs, and two PDs. Target lesion responses were three PRs, six SDs out of nine subjects. Overall effective response (PR + SD) was 78%. The median PFS was 6.3 months, whereas the median OS was 15.5 months. Two subjects from Cohort 1 and 2 showed down staging, being reevaluated as resectable cancer, and finally achieved surgical CR (Table 3).

**Table 3:** Efficacy evaluation

Patient no.		Evaluation (RECIST ver 1.1)			Time to response (days)	Duration of response (days)	PFS (days)	OS (days)
		Target response	Overall response	Surgical response				
Cohort 1	HF-1-02	SD	PD				119	150
	HF-1-04	SD	PD				91	465
	HF-1-05	PR	PR	CR	48	288	335	611
Cohort 2	HF-2-01	SD	SD				663	1211
	HF-2-02	PR	PR	CR	13	444	456	1189
	HF-2-03	SD	SD				48	336
Cohort 3	HF-3-02	SD	SD				217	694
	HF-3-03	SD	SD				69	273
	HF-3-04	PR	PR		34	156	189	255

*PFS* progression free survival, *OS* overall survival, *PR* partial response, *SD* stable disease, *CR* complete response

### Cases with surgical CR

**Fig. 2:**
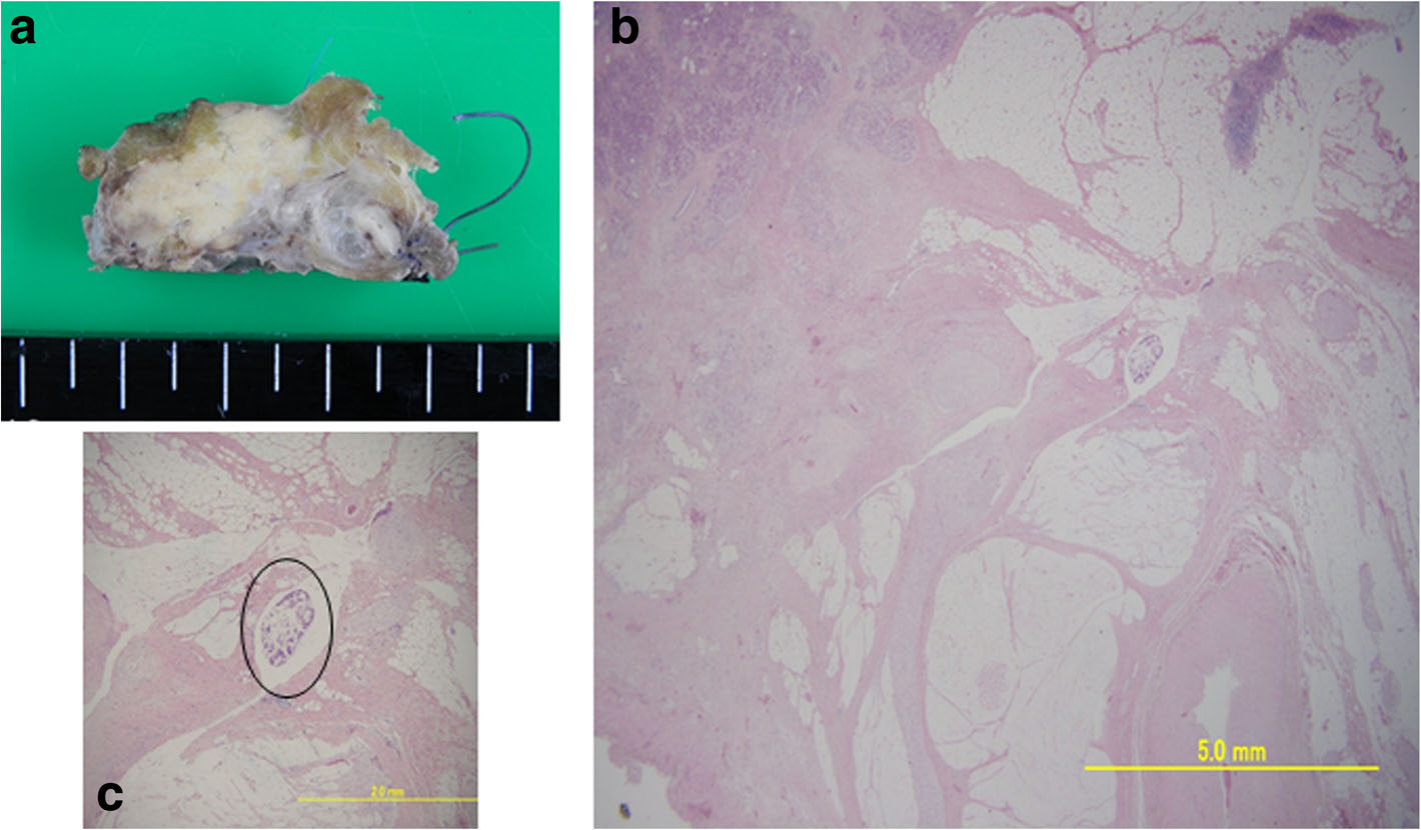
**a** A cut surface of the pancreatic body showed a fibrotic tissue in the area where the tumor was located (HF-1-05). **b** On histological analysis, 99% of the cancer cells had disappeared and had been replaced with fibrotic tissue. **c** High-power photomicrograph revealed a minute residual cancer tissue (circle)

**Fig. 3:**
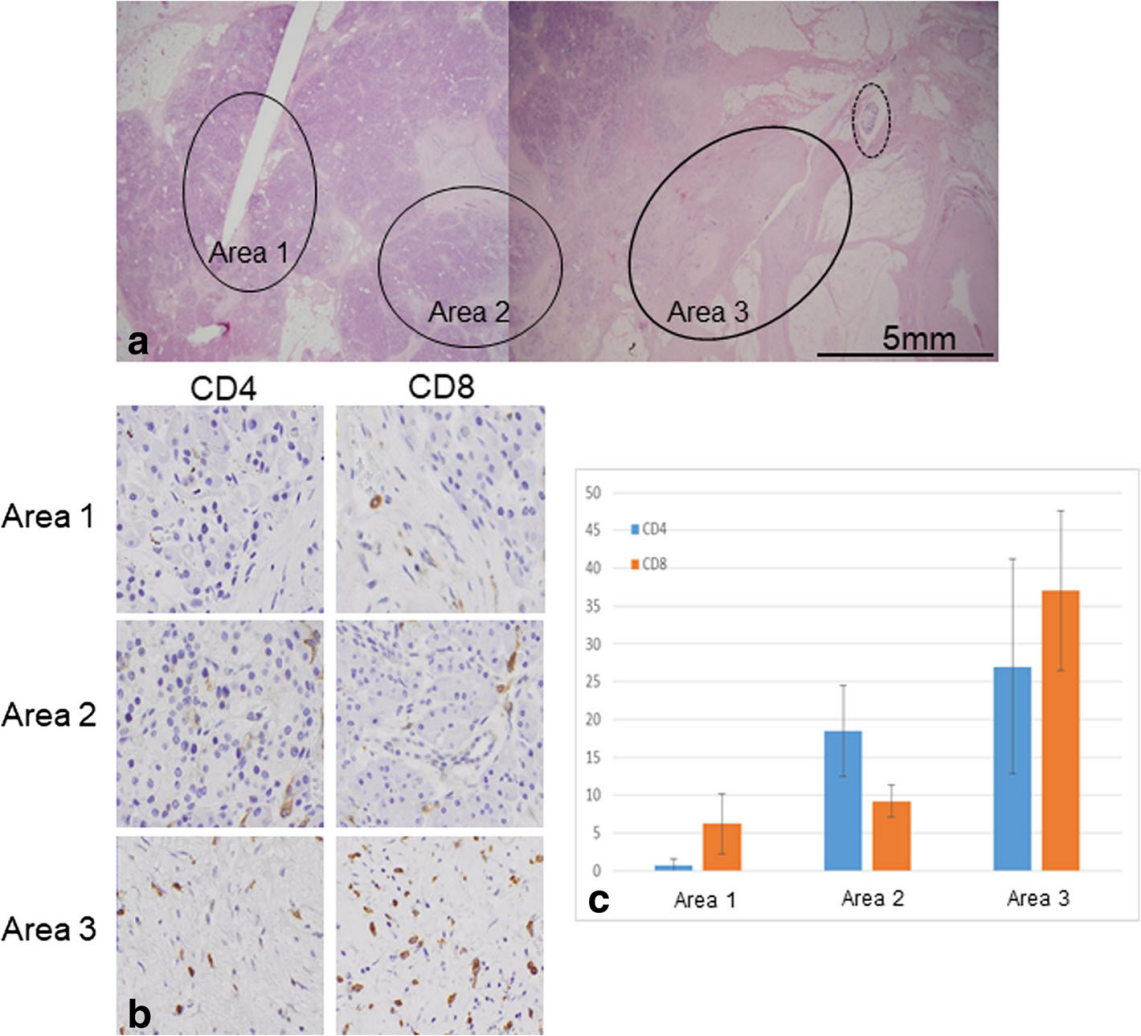
Evaluation of CD4+ and CD8+ cells infiltration around the cancer tissue (HF-1-05). **a** Three areas in different distances (circle) from the residual cancer (dot-line circle) were evaluated. **b**, **c** Infiltration of CD4+ and CD8+ cells was significant in the fibrosis near the residual cancer tissue (area 3) and it became obscure as the areas receded from the cancer tissue

**Fig. 4:**
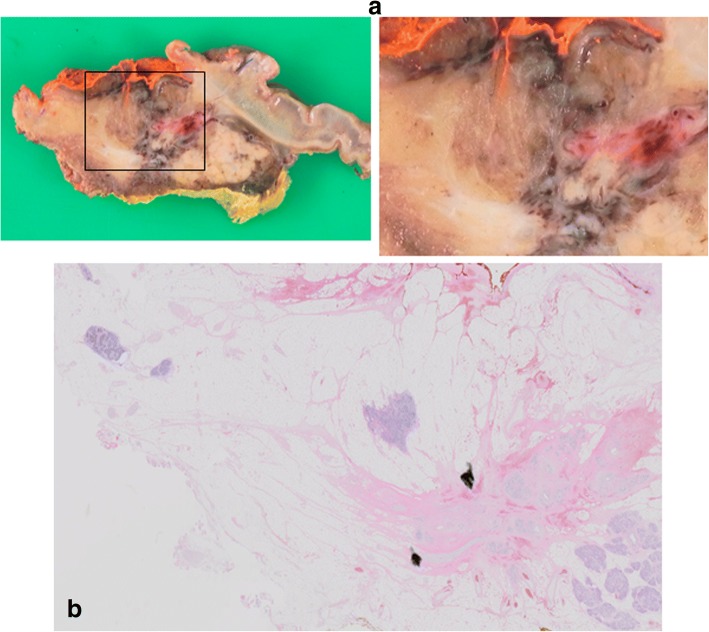
**a** A cut surface of the pancreatic head of HF-2-02. The left image showed showed fibrosis in the middle and the right showed a magnified image. **b** Histopathological findings of the tumor in the pancreatic head showed 90% disappearance of cancer cells with fibrosis

**Fig. 5:**
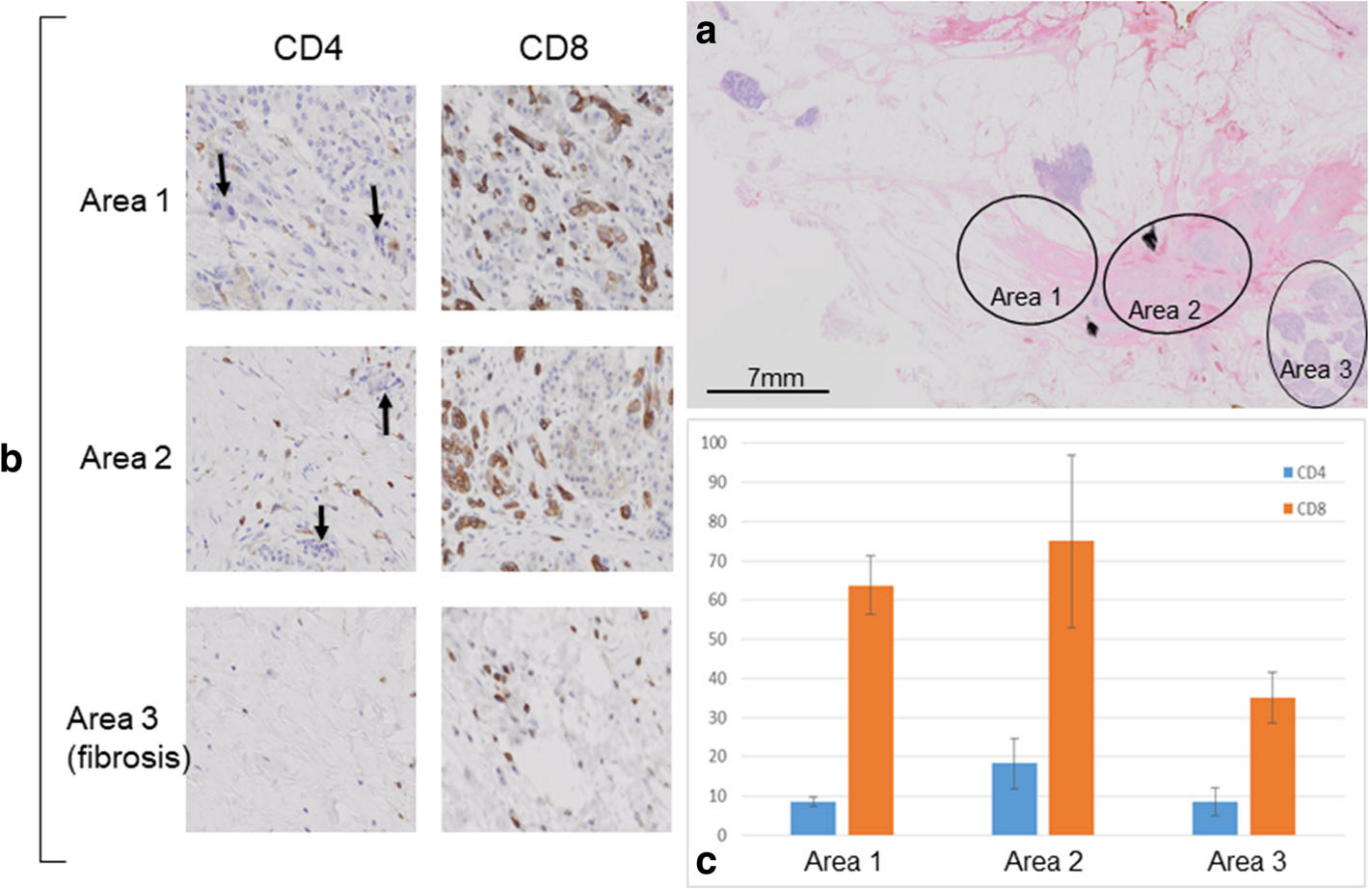
Evaluation of CD4+ and CD8+ cells infiltration around the cancer tissue (HF-2-02). **a** Three different areas (circle) were evaluated. **b**, **c** High-power photomicrograph showed diffuse persistence of cancer cells (arrow), and infiltration of CD8+ cells was detected along by the cancer cells

The first case was a 66 years old female in Cohort 1 who received HF10 of 1.0 x 10^6^pfu × 4 times and had radiation therapy of 1.8 Gy × 28 times after clinical trial. Distal pancreatectomy was performed 5 months after registration and the resected specimen showed 99% disappearance of the cancer cells in the tumor (Fig. 2). We examined the infiltration of CD4+ and CD8+ cells by immunostaining. Infiltration of CD4+ and CD8+ cells was significant in the fibrosis near the residual cancer cells and it became obscure as the areas receded from the cancer cells (Fig. 3). Unfortunately, she developed peritoneal dissemination 6 months after surgery and the survival time was 22 months. The second case was a 65 years old male in Cohort 2 who received HF10 of 3.0 x 10^6^pfu × 4 times. This patient had radiation therapy of 1.8 Gy × 28 times before clinical trial. The invasion to plexus of superior mesenteric artery had shown decrease in size after HF10 injection, and he underwent pancreaticoduodenectomy 7 months after registration. Resected specimen showed 90% disappearance of cancer cells (Fig. 4). On immunostaining, infiltration of CD8+ cells was detected alongside the cancer cells (Fig. 5). Although CT scan revealed the recurrence in mesenteric lymph nodes 6 months after the surgery, long term survival was obtained and the survival time was 39.6 months.

## Discussion

In this study, we performed a phase I dose-escalation trial of HF10 direct injection therapy for unresectable locally advanced pancreatic cancer under EUS-guidance in combination with erlotinib and gemcitabine administration, which was safe and effective with all doses (1 × 10^6^, 3 × 10^6^, or 1 × 10^7^ pfu/day × 4 times).

Since 1997, gemcitabine therapy has been the standard first-line treatment for patients with unresectable locally advanced or metastatic pancreatic cancer with a median survival rate of 4.4–5.6 months [15, 16]. Several combination treatments based on gemcitabine have been investigated; however, most have not significantly improved survival versus gemcitabine alone [17–29], except for the combination therapy with gemcitabine plus elrotinib, which showed a significant improvement in overall survival for 2 weeks in median [30]. More recently, FOLFIRINOX therapy (leucovorin, fluorouracil, irinotecan and oxaliplatin) [31] and gemcitabine plus nab-paclitaxel therapy [32] have been approved for unresectable pancreatic cancer in Japan. They have significantly improved survival and are now used as a first-line chemotherapy for unresectable pancreatic cancers. However, since the majority of patient eventually progress on these therapies, novel therapies are required.

HF10 has shown a promising antitumor effect with a high safety margin in the investigator-initiated clinical studies for pancreatic cancer [5]. Phase I clinical trial using HF10 in advanced pancreatic cancer was reported from the department of surgery II at Nagoya University in Japan [10]. They initiated pilot studies by injecting six patients with non-resectable pancreatic cancer with three doses of HF10 (1 × 10^5^/two patients, 5 × 10^5^/one patient, and 1 × 10^6^/three patients). They observed some therapeutic potential based on tumor marker levels, survival, pathological findings and diagnostic radiography. The important thing is that there were no adverse side-effects in these patients. As gemcitabine is an anticancer drug which has been well investigated in combination with many OVs in different malignancies including pancreatic cancer [33–37], the combination of HF10 and gemcitabine can be an ideal therapy against pancreatic cancer to achieve a potent antitumor effect with minimal side effects. Given the results in a Japanese phase II pancreatic cancer trial using gemcitabine and erlotinib with acceptable tolerance and mild AEs [38], we have decided to combine HF10 with gemcitabine and erlotinib in our study. Five out of 10 patients showed Grade III myelosuppression and one patient showed interstitial pneumonia after treatment, but all of them recovered by discontinuing the treatment. Unfortunately, two patients developed serious AEs (perforative peritonitis and hepatic dysfunction). Regarding the perforative peritonitis of HF-1-02, the tumor radiologically showed direct invasion to the duodenum from the beginning. Eventually the tumor caused obstruction of the duodenum with increased pressure inside the lumen, which led to the perforation. As a result, all of the AEs occurred in our study were judged as AEs unrelated to HF10, and there was no increase in AEs according to the dose of HF10 escalating up to 1 × 10^7^ pfu/day x 4times.

It is noteworthy that although the median PFS in our study was relatively short as 6.3 months, median OS was 15.5 months and two patients achieved long term survival over 3 years. Interestingly, the patient who achieved the longest survival did not have surgery and the best overall response was SD, suggesting the development of acquired immunity by this treatment. With regard to the histopathological findings, previous clinical studies have revealed that HF10 increased the number of CD4+, CD8+ and natural killer cells within the tumor, which may lead to the tumor growth reduction and prolonged survival rates [5, 7, 8]. In two cases who underwent surgery in our study, infiltration of CD4+ or CD8+ cells was well detected in the area nearby the residual cancer cells. It is considered that the anti-tumor effects of OVs are not only the direct cancer cell destruction but also the stimulation of anti-tumor immunity, and these results support the above hypothesis.

## Conclusions

HF10 direct injection therapy for unresectable locally advanced pancreatic cancer under EUS-guidance in combination with erlotinib and gemcitabine administration was safe and demonstrated anti-tumor effectiveness with higher those than used in previous studies. HF10 combination therapy should be explored further in large prospective studies. In the near future, we plan to perform a clinical trial in combination of HF10 and gemcitabine with nab-paclitaxel treatment aiming at unresectable pancreatic cancers with or without metastases.

## Additional file

Additional file 1: **Table S1.** Criteria Defining Resectability Status. (DOCX 18 kb)

## Abbreviations

AE: Adverse event
CR: Complete response
CT: Computed tomography
CTCAE: Common Terminology Criteria for Adverse Events
DLT: Dose limiting toxicity
ECOG: Eastern Cooperative Oncology Group
EUS: Endoscopic ultrasound
EUS-FNA: EUS-guided fine needle aspiration
HNSCC: Head and neck squamous cell carcinoma
HSV-1: Herpes simplex virus-1
MTD: maximum tolerated dose
OS: Overall survival
OV: Oncolytic virus
PD: Progressive disease
PFS: Progression free survival
PR: Partial response
PS: Performance status
RECIST: Response Evaluation Criteria in Solid Tumors
SD: Stable disease
ULN: Upper limits of normal
