# Supplementary material for: A Phase I clinical trial of EUS-guided intratumoral injection of the oncolytic virus, HF10 for unresectable locally advanced pancreatic cancer
Source: BMC Cancer. 2018 May 25;18:596. doi: 10.1186/s12885-018-4453-z (PMC5970460; doi:10.1186/s12885-018-4453-z)
Supplement: Supplementary file 1 — Table S1. Criteria Defining Resectability Status. (DOCX 18 kb) [file 12885_2018_4453_MOESM1_ESM.docx]

| Table S1. Criteria Defining Resectability Status | |  |
| --- | --- | --- |
|  |  |  |
| Resectability  Status | Arterial | Venous |
| Resectable | No arterial tumor contact (celiac axis [CA], superior mesenteric artery) [SMA], or common hepatic artery [CHA]). | No tumor contact with the superior mesenteric vein (SMV) or portal vein (PV) or ≦180° contact without vein contour irregularity. |
| Borderline  Resectable | Pancreatic head/uncinate process: ・ Solid tumor contact with CHA without extension to celiac axis or hepatic artery bifurcation allowing for safe and complete resection and reconstruction. ・ Solid tumor contact with the SMA of ≦180° ・ Solid tumor contact with variant arterial anatomy (ex: accessory right hepatic artery, replaced right hepatic artery, replaced CHA, and the origin of replaced or accessory artery) and the presence and degree of tumor contact should be noted if present as it may affect surgical planning. Pancreatic body/tail: ・ Solid tumor contact with the CA of ≦180° ・ Solid tumor contact with the CA of > 180°without involvement of the aorta and with intact and uninvolved gastroduodenal artery thereby permitting a modified Appleby procedure [some members prefer this criteria to be in the unresectable category]. | ・ Solid tumor contact with the SMV or PV of > 180°, contact of   ≦180°with contour irregularity of the vein or thrombosis of the  vein but with suitable vessel proximal and distal to the site of  involvement allowing for safe and complete resection and vein  reconstruction.  ・ Solid tumor contact with the inferior vena cava (IVC). |
| Unresectable | ・ Distant metastasis (including non-regional lymph node metastasis) Head/uncinate process: ・ Solid tumor contact with SMA > 180° ・ Solid tumor contact with the CA > 180° ・ Solid tumor contact with the first jejunal SMA branch Body and tail ・ Solid tumor contact of > 180°with the SMA or CA ・ Solid tumor contact with the CA and aortic involvement | Head/uncinate process ・ Unreconstructible SMV/PV due to tumor involvement or occlusion (can be due to tumor or bland thrombus) ・ Contact with most proximal draining jejunal branch into SMV Body and tail ・ Unreconstructible SMV/PV due to tumor involvement or  occlusion (can be due to tumor or bland thrombus) |
